# Owners’ Beliefs regarding the Emotional Capabilities of Their Dogs and Cats

**DOI:** 10.3390/ani13050820

**Published:** 2023-02-24

**Authors:** Olivia Pickersgill, Daniel S. Mills, Kun Guo

**Affiliations:** 1 Department of Life Sciences, University of Lincoln, Lincoln LN6 7DL, UK; 2 School of Psychology, University of Lincoln, Lincoln LN6 7DL, UK

**Keywords:** emotion, expression, dog, cat, owner perception, welfare

## Abstract

**Simple Summary:**

Understanding how pet dogs and cats are feeling is very difficult and getting it wrong could result in welfare issues for the animals and the risk of injury for humans. Scientific research on pet emotion is in its early stages and pet owners are currently one of the best sources of information because they spend so much time with their animals. In this online survey, 438 owners were asked whether their dogs and/or cats could express 22 different emotions. If they answered ‘yes’, they were then asked how they identify that emotion in their pet. Owners believed dogs could feel more emotions than cats, and that they could use different sets of behavioral signs to identify different dog/cat emotions. The number of reported dog emotions tended to increase with the owner’s increased personal experience with dogs but decreased with the owner’s increased professional experience with dogs. Owners who owned both cats and dogs believed that cats could feel fewer emotions than owners that owned only cats. These owner perceptions are useful in advancing research in the field of animal emotions as they provide a starting point for validating each emotion in these species.

**Abstract:**

The correct interpretation of an animal’s emotional state is crucial for successful human–animal interaction. When studying dog and cat emotional expressions, a key source of information is the pet owner, given the extensive interactions they have had with their pets. In this online survey we asked 438 owners whether their dogs and/or cats could express 22 different primary and secondary emotions, and to indicate the behavioral cues they relied upon to identify those expressed emotions. Overall, more emotions were reported in dogs compared to cats, both from owners that owned just one species and those that owned both. Although owners reported a comparable set of sources of behavioral cues (e.g., body posture, facial expression, and head posture) for dogs and cats in expressing the same emotion, distinct combinations tended to be associated with specific emotions in both cats and dogs. Furthermore, the number of emotions reported by dog owners was positively correlated with their personal experience with dogs but negatively correlated with their professional experience. The number of emotions reported in cats was higher in cat-only households compared to those that also owned dogs. These results provide a fertile ground for further empirical investigation of the emotional expressions of dogs and cats, aimed at validating specific emotions in these species.

## 1. Introduction

Dogs and cats may provide a wide range of health, emotional, behavioral, cognitive, educational, and social benefits to humans, and support economic growth [1,2,3]. To maximize these animals’ social and economic value, we need to understand and interpret their emotions appropriately so that high-quality human–animal interaction and animal welfare can be achieved. A misinterpretation can lead to suffering in these animals (e.g., behavioral problems), owner distress and mental health issues, reduced societal benefits, and possible physical harm to humans [4,5]. For instance, dog bites often occur because humans cannot correctly identify the early signs of discomfort or distress in dogs [6,7]. Many dog owners also believe their dogs can feel guilt [8], yet studies have shown that so-called ‘guilty’ behavior exhibited by dogs after a ‘prohibited’ behavior may instead be linked to owners’ scolding behavior [9]. If the dog cannot understand why their owner is scolding them, it could cause them to feel unnecessary distress and negatively impact its welfare.

Considering the importance of understanding emotion in human–animal interactions, we know surprisingly little about what extent dogs and cats express a typical range of emotions and what behavioral signs are indicative to humans for potentially detecting these emotions. This lack of information may be due to inherent challenges in studying emotion in nonhuman animals, as their behavior and communicative signals vary greatly from our own and we cannot rely on language to help bridge the gap. Furthermore, human emotion perception and processes seem to be preferentially adapted to conspecifics, but we may adopt an identical or similar process to perceive and interpret both conspecific and heterospecific emotional cues [10]. For instance, humans displayed qualitatively similar gaze distributions by applying the same local facial features when viewing human and dog faces [11] or judging the approachability of these faces [12], and by gazing predominantly at the faces when assessing whole-body human and dog emotional expressions [13]. Considering that humans and dogs facially express the same emotion differently [14] and many non-facial cues (e.g., tail movements, ear and body postures) are informative indicators of dog affective states [15,16] but are less evident or absent in humans, the same gaze strategy, which is adapted for the effective perception of human emotions, may not function well in perceiving dog emotions, subsequently leading to poor human performance in recognizing dog emotions [10,13,17]. 

However, regardless of their ability or performance in identifying animal emotions, pet owners are arguably an important potential data source for studying companion animal emotions because of their intensive interaction with their pets, their experience of emotional triggers and contexts associated with specific pet emotions, and their knowledge or sensitivity to subtle pet emotional behaviors. Although this data source is naturally subjective and susceptible to anthropomorphic bias (i.e., attributing human emotion characteristics to animals), owner perception of pet emotions may provide consistent trends and collective evidence that could provide a fertile source for guiding further empirical research to determine whether these owner observations are accurate.

To date, only a few studies have examined owners’ beliefs about their pet’s emotions, with large variances between individual owners’ reports and between studies. Generally, dog and cat owners believed their pets could express a wide range of emotions, which can be grouped into primary/basic and secondary/complex emotions [8,18,19,20]. Primary emotions (e.g., anger, disgust, fear, joy, sadness, and surprise) are more widely supported in animals as they are viewed as evolutionarily adaptive and biologically predisposed [21]. In contrast, the occurrence of secondary emotions, which can be a mixture of primary emotions and may embrace both positive and negative effects concurrently alongside cognitive elaborations (e.g., compassion, disappointment, embarrassment, empathy, envy, and pride) [22], are considered more controversial. This is usually because many of these secondary emotions involve meta-cognitive (e.g., cognitive appraisal of own and/or other’s mental states, such as jealousy) and/or self-conscious evaluative processes (e.g., evaluation of own behavior against learned and internalized rules or standards, such as guilt or shame) that are often believed to be beyond the representational capacities of human infants and nonhuman animals [23]. Nevertheless, roughly half of the pet owners reported some secondary emotions in their pets [8,18].

Furthermore, dog and cat owners may report differently about their pet’s emotions. In a recent survey, participants attributed primary emotions to both dogs and cats roughly equally (88% dogs vs. 87% cats); however, secondary emotions were only reported by 44% of cat owners, compared to 55% of dog owners [8]. Furthermore, cats were more frequently attributed the emotions of anger and disgust, while all other emotions surveyed were reported more frequently in dogs (also see [19]). However, these studies mainly used a ‘between-subject’ design in which participants were asked to report the pet they have lived with the longest, even if they owned both dogs and cats. It is unclear whether these findings could be extended to those people with experience and knowledge of the emotional behavior of both dogs and cats. 

Regarding the large variances in the reported number and type of emotions across dog and cat owners, this might be caused by both individual differences in owners and breed differences in dogs/cats. According to recent studies, the number of owner-reported pet emotions or the accuracy in recognizing pet emotional expression images could be affected by an owner’s gender and age [19,24], cultural background [25], mental health condition (e.g., anxiety and depression) [26,27], personal or professional experience with the species [24,28], and attachment with the species [29]. Additionally, wide variations in morphology across dog and cat breeds, such as coat color, coat length, ear shape, and tail shape [24,29,30,31], could affect the visibility of animal facial and/or bodily expressions and subsequently owners’ abilities in reading these animal emotions. However, no owner perception studies to date have collated all of these owner and pet breed factors to assess their potential impact on the identification of animal emotions.

To address these potential confounding factors in previous research, in the current study we aimed to directly compare the number of emotions reported by dog and cat owners, both in homes with only one species and homes with both species, using an extended emotion list including primary and secondary emotions commonly reported in both human and animal emotion research e.g., [8,18,32]. To our knowledge, it is the first study to collect data on multiple species from the same owners. If significant differences are found, a detailed analysis could reveal which individual emotions are driving the variation. Based on previous research [8,18,19,20], we hypothesized that more emotions (especially secondary emotions) would be reported in dogs than in cats, and this trend would be qualitatively similar for owners that keep both species.

We also requested owners to indicate the common animal arousal, signaling, and behavioral tendency signs used by them to detect specific animal emotions [18,33]. Given that human primary and secondary emotional expressions are associated with distinctive patterns of dynamic multistage and multimodal behavior (including facial action, head/body gesture, movements, vocalization, etc.) [32,34], it is plausible that different dog/cat emotional expressions could also be differentiated based on the information contained within these behavioral changes. Our final aim was to collect data containing a large group of owners (e.g., varying in age, gender, and pet experience) with pets varying in age, sex, and morphology so that we could determine those factors which may influence the number of emotions reported within a species.

## 2. Materials and Methods

### 2.1. Participants

Dog and cat owners over 16 years old were mainly recruited via social media (Facebook, Instagram, and LinkedIn). A recruitment poster was uploaded to the authors’ personal social media pages as well as relevant Facebook group pages (found via searches for ‘dog owners’, ‘cat owners’, ‘dog’, and ‘cat’). In addition to social media, some participants were recruited via dog training and behavior businesses. The survey was open for 6 weeks and there was no incentive for participation. To minimize anthropomorphic attribution, participants had to have lived with their pets for at least two years so that they could answer the survey questions by using their direct and intimate experience with their pets rather than their general ideas about animal emotions [8]. Participants that had both a dog and a cat were asked to complete the survey for both species. Participants that had multiple dogs/cats could choose to complete the survey multiple times if their pets were different breeds. Prior to the study, the research purpose and survey tasks had been explained to the participants, and informed consent was obtained from each of them. All procedures complied with the British Psychological Society Code of Ethics and Conduct. The study was approved by the Ethical Committee of the University of Lincoln (2022-1069).

### 2.2. Questionnaires

The online survey was created in Qualtrics and included three sections: (1) owner demographic information, (2) pet demographic information, and (3) an emotion questionnaire. Owner demographic questions were a mixture of free-type and multiple choice and included questions on age, gender, cultural background, years of pet ownership, professional experience with dogs/cats (e.g., trainer, behaviorist, groomer, kennel/cattery worker, dog walker, dog/cat sitter, rescue work, fosterer, education), and mental health information (e.g., autism, anxiety, and depression). Pet demographic questions were also a mixture of free-type and multiple choice (with picture guides for dog ear and muzzle shape), and included age, sex, breed, and morphological characteristics (for dogs, they included fur color, muzzle size (short, medium, and long), ear shape (cropped, pricked/bat, drop/folded, V-shaped, rose-shaped, and semi-pricked/button), and tail shape (docked/bobbed, tightly curled over back, and other); for cats, they included fur color, fur length (hairless, short, and long), fur pattern (tabby, solid, bi-color, tortoiseshell/calico, and colorpoint), ear shape (pricked, curled, and folded), and tail shape (no tail, bobbed, curly, other)). The emotion questionnaire included 6 primary emotions (anger, disgust, fear, joy, sadness, and surprise) and 16 secondary emotions (amusement, anxiety, boredom, confusion, curiosity/interest, disappointment, embarrassment, empathy, frustration, grief, guilt/shame, jealousy, love/affection, pain, positive anticipation, and pride). Owners were asked whether their pet could express a certain emotion (Yes/No). If the owners answered yes to a particular emotion, they were then asked to choose the behavioral cues they used to identify this emotion. They chose from a list of behavioral cues that included proximity to the owner, facial expression, head posture, ear posture, body posture, tail height, tail wagging, vocalization, eye contact, speed of movement, physical contact and action, and “others” (adapted from [18]), and could select as many as they deemed applicable.

### 2.3. Data Analysis

All data analyses were conducted in R 4.1.2. Linear models were used to analyze whether the number of owner-reported animal emotions (i.e., the total number of reported emotions, number of primary emotions, and number of secondary emotions) varied between dogs and cats, both in dog/cat-only homes and in homes with both species. For each emotion, binary generalized linear models were then used to compare the percentage of ‘yes’ and ‘no’ responses reported for dogs and for cats (dogs in dog-only homes vs. cats in cat-only homes, dogs vs. cats in homes that owned both species).

Furthermore, multifactor linear models (one for owner factors and one for pet factors) were used to assess whether individual differences in owners and breed differences in dogs/cats could significantly alter the total number of emotions reported for dogs or cats. For owners that had professional experience with dogs or cats, their professions were categorized to facilitate post hoc comparisons (all other data were analyzed in their original form). Specifically, trainers and behaviorists were grouped together, all veterinary professional staff were grouped together, and the remaining respondents were divided into ‘hands-on’ (e.g., kennel/cattery workers, rescue workers, groomers, and pet sitters) and ‘hands-off’ groups (e.g., education and research). Binary generalized linear models were used to analyze the proportion of ‘yes’ and ‘no’ responses for each emotion in cat/dog-only vs. cat and dog homes to understand which emotions were driving the results of the owner multifactor linear model.

## 3. Results

In total, 453 completed survey responses were collected. Fifteen were excluded from further analysis because the respondents had lived with their pets for less than 2 years, leaving 438 responses to be analyzed. Of these, 277 were from dog-only owners, 93 were from cat-only owners, and 68 were from owners of both species. No respondents completed the survey for multiple dogs or cats of different breeds.

### 3.1. Do Owners Believe Dogs Have More Emotions than Cats?

**Figure 1:**
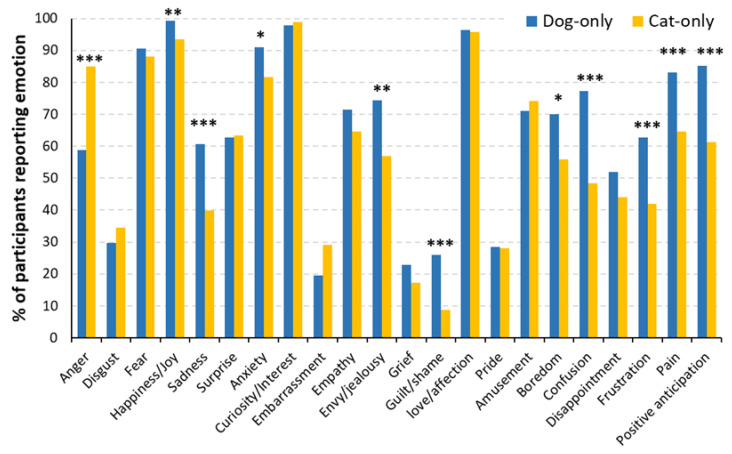
Percentage of participants reporting individual emotions as a function of species in dog-only (*n* = 277) and cat-only (*n* = 93) homes. * *p* < 0.05, ** *p* < 0.01, *** *p* < 0.001.

Dog-only owners reported a significantly higher number of emotions than cat-only owners (*F*(1, 368) = 12.65, *p* < 0.001; Figure 1). On average, 65% of dog owners and 58% of cat owners believed their pets could express a given emotion. Regardless of species, the five most frequently reported animal emotions were curiosity (98%), happiness (96%), love (96%), fear (89%), and anxiety (86%); the five least reported emotions were guilt (17%), grief (20%), embarrassment (24%), pride (28%) and disgust (32%). There also existed significant variations in individual emotions reported between dog and cat owners. Specifically, for primary emotions, happiness and sadness were reported significantly more frequently in dogs, whereas anger was reported more frequently in cats (all *p*-values < 0.05). For secondary emotions, anxiety, boredom, confusion, envy/jealousy, frustration, guilt/shame, pain, and positive anticipation were reported more frequently in dogs (all *p*-values < 0.05). All other emotions were reported with comparable frequencies between dog and cat owners (all *p*-values > 0.05).

**Figure 2:**
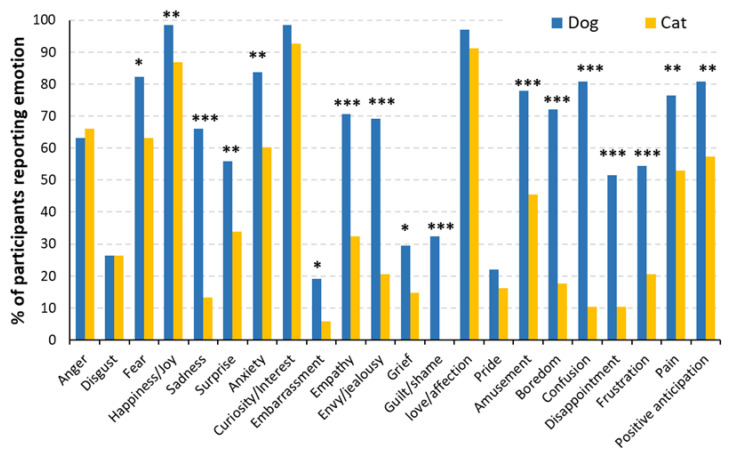
Percentage of participants reporting individual emotions as a function of species in homes with both dogs and cats (*n* = 68). * *p* < 0.05, ** *p* < 0.01, *** *p* < 0.001.

Respondents that owned both cats and dogs also reported significantly more emotions in their dogs (*F*(1, 134) = 83.54, *p* < 0.001; Figure 2). Unlike the dog-only versus cat-only comparison, these owners consistently reported more dog expressions for both primary (*F*(1, 134) = 20.21, *p* < 0.001) and secondary emotions (*F*(1, 134) = 99.62, *p* < 0.001). Referencing the 6 primary emotions, owners reported fear, happiness, sadness, and surprise more frequently in their dogs than in their cats (all *p*-values < 0.05). Almost all secondary emotions were reported significantly more frequently in dogs (all *p*-values < 0.05), except for curiosity, love, and pride (all *p*-values > 0.05).

### 3.2. Do Owners Rely on the Same Cue Sources to Identify Dog and Cat Emotions?

Table 1 compares common cue sources used by over 50% of respondents to identify a given emotion expressed by dogs and cats. Across all emotions, body posture, facial expression, and head posture were the three most frequent sources of cues used by both dog and cat owners (reported by 66% vs. 59% vs. 52% of dog owners, and 63% vs. 47% vs. 43% of cat owners). Although there existed quantitative differences in the reported frequency for a given source of information, dog and cat owners used some common sources to detect 16 out of 22 animal emotions and relied on the same set of sources in identifying 5 emotions (disgust, empathy, pride, confusion, and frustration; Table 1). However, when differentiating (at least some) different emotions in either dogs or cats, a distinctive combination of sources of behavioral cues tended to be informative for owners (e.g., facial expression and body posture for dog embarrassment vs. proximity to the owner and physical contact for dog empathy), and this tendency was more evident for dog owners than cat owners. Furthermore, there were no clear differences in common sources of behavioral cues (e.g., up to three most frequently reported cues) used to detect different animal emotions between dog-only owners and dog + cat owners or between cat-only owners and cat + dog owners.

### 3.3. Which Owner or Pet Factors Affect the Number of Reported Animal Emotions?

**Figure 3:**
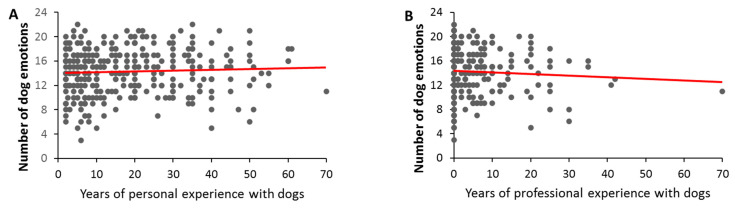
The number of emotions reported in dogs by owners with varying levels of personal (**A**) and professional experience with dogs (**B**). Trendlines show the direction of significance.

The analyzed owner factors included owner’s age (1% of owners were 16–20 years old, 30% were 20–29, 21% were 30–39, 17% were 40–49, 18% were 50–59, 11% were 60–69, and 3% were over 70), gender (87% female, 12% male, and 1% nonbinary), cultural background (90% Caucasian, 3% Asian, 0% African, 4% others, and 3% preferred not to say), mental health profile (24% with autism, anxiety, depression, etc., 72% none, and 4% preferred not to say), years of pet ownership, years of professional experience with dogs/cats, type of dog-/cat-related profession, and pet species (Table 2). For dogs, the number of reported emotions (varying between 3 and 22) was only affected by the years of experience owners had, both in a personal (varying between 2 and 70 years; *F*(1, 269) = 6.87, *p* = 0.009) and professional (varying between 0 and 70 years, *F*(1, 269) = 7.35, *p* = 0.007) capacity. Specifically, the number of dog emotions reported tended to increase with increasing years of personal experience (Figure 3A) but decreased with increasing professional experience (Figure 3B). These patterns appear quite weak visually (Figure 3) and are not significant with a simpler Pearson correlation analysis (personal experience: *r* = 0.05, *p* = 0.17; professional experience: *r* = −0.06, *p* = 0.13). 

**Figure 4:**
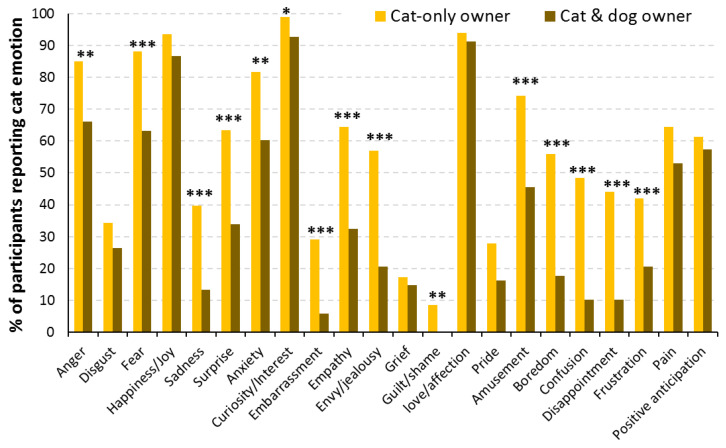
Percentage of participants reporting individual emotions in their cats, comparing cat-only homes (*n* = 93) with those that also own dogs (*n* = 68). * *p* < 0.05, ** *p* < 0.01, *** *p* < 0.001.

For cats, the number of reported emotions was only affected by pet species owned (i.e., whether owners also owned dogs; *F*(1, 84) = 28.87, *p* < 0.001; Figure 4). Cat-only owners reported more emotions in their cats compared to owners of both cats and dogs (*F*(1, 159) = 54.73, *p* < 0.001). This pattern occurred for both primary (*F*(1, 159) = 31.53, *p* < 0.001) and secondary emotion categories (*F*(1, 159) = 53.39, *p* < 0.001). Specifically, compared to owners of both species, cat-only owners more frequently reported the primary emotions of anger, fear, sadness, and surprise, and secondary emotions of anxiety, curiosity, embarrassment, empathy, envy/jealousy, guilt, amusement, boredom, confusion, disappointment, and frustration (all *p*-values < 0.05).

Our analyzed pet factors included pet age, sex, breed, ear shape, tail shape, dog muzzle shape, dog coat color, cat coat length, and cat coat pattern (Table 3 and Table 4). The multifactor linear model analysis revealed that the number of reported dog or cat emotions was not affected by any of these factors (all *p*-values > 0.05).

## 4. Discussion

The three objectives of this exploratory study were to (1) directly compare dog and cat owners’ beliefs about the number of emotions their pets could express, (2) evaluate what behavioral changes were associated with each emotional expression by owners, and (3) analyze the influence of various owner and pet-related factors on the owners’ belief. Regarding the 1st objective, our analysis revealed that pet owners only reported more anger in cats than in dogs and a more frequent expression of two other primary and eight secondary emotions in dogs (Figure 1). The latter trend was exaggerated in owners of both dogs and cats in which dogs were believed to express 4 primary and 13 secondary emotions more frequently than cats (Figure 2). Such variation in the perceived pet emotions between dogs and cats may partly be due to differences in the emotional bond between owners of the two species, as owners of both dogs and cats have reported a greater level of emotional closeness with their dogs than their cats [20,35]. Cat owners were also less likely to consider their cats as ‘family members’ than dog owners, indicating a lower level of owner–cat attachment than owner–dog attachment [19]. However, caution in making such generalizations is warranted as recent work has indicated a wide disparity in the nature of cat–owner relationships, with some owners forming very strong, emotionally dependent relationships [36]. Our novel observation of the exaggerated belief of dog emotion capabilities (especially) from owners of both dogs and cats may further suggest the crucial role of human–pet attachment in perceiving pet emotions. Future work could consider the effect of the nature of the pet–owner relationship on the number of perceived emotions of a pet.

Nonetheless, domestication history may also be important. Dogs were historically selected to work for humans, in roles typically requiring frequent heterospecific communication and understanding [20]. Cats, on the other hand, had a more commensal relationship with humans, typically hunting rodents independently [37]. As semi-solitary animals, their need for heterospecific communication and the ability to express emotion in a way that others can easily interpret may not have been as evolutionarily important [19]. Cats also tend to look at their owners less frequently [38]. Owners may thus perceive cats’ behaviors as generally less sociable and communicative or simply independent [39], all of which could reduce the level of perceived emotion in this species by owners.

The fact that cats are semi-solitary animals may also explain why anger was the only emotion reported significantly more frequently in cats than in dogs by cat-only owners. For instance, aggressive behavior may be more common in cat-only households with multiple cats competing for key resources and territorial boundaries [40]. Cat-only owners may also tend to spend more time interacting with their cats compared to owners of both species, which could increase the prevalence of petting-related aggressive incidents [41]. These possibilities could be addressed in future research by more in-depth and structured interviews with cat and dog owners, yielding a more detailed understanding of processes underlying those believed variations in emotional capability between the two species.

Regarding the second objective, our analysis revealed that both dog and cat owners tended to rely on a comparable set of sources of behavioral cues, especially body posture, facial expression, and head posture, to detect their pet’s emotions, indicating the important role of nonfacial cues in identifying animal emotions. As the facial expression is generally the dominant channel of emotional expression in human–human emotional perception [42], humans predominantly rely on facial cues to recognize other humans’ emotions and tend to adopt a similar strategy to detect animal emotion by mainly gazing at the faces when assessing whole-body animal emotional expressions [13]. This strategy, adapted for the effective perception of human emotions, may lead to poor human performance in recognizing dog emotions [10,13,17]. This is because some nonfacial cues (e.g., ear and body posture) are highly informative indicators of dog emotional states [15,16]. Indeed, the experienced pet owners (≥2 years of pet ownership) in this study consistently reported body posture and head posture as two of the most frequently used behavioral cues to decipher their pet’s emotions, indicating the adoption of a learned and animal-specific strategy in perceiving pet emotions. It would be informative to objectively measure, in future research, the effectiveness of these owners’ strategies in recognizing different animal emotions, especially from unfamiliar dogs/cats. 

The pet (especially dog) owners further believed that many different categories of animal emotions were associated with distinctive combinations of behavioral cues, such as changes in facial expression and body posture for dog embarrassment, and changes in proximity to the owner and physical contact for dog empathy (Table 1). These beliefs are consistent with previous empirical observations of dogs displaying distinctive facial movements, coded through an objective, anatomically based dog facial action coding system in response to different categories of emotional triggers [14]. Hence, it is plausible that, like humans [32,34], dogs can display a distinctive range of primary and secondary emotions that are recognizable through emotion-specific dynamic changes in facial and bodily expressions. This hypothesis could also be empirically examined in future research.

Regarding the third objective, our analysis revealed that the number of reported dog emotions had a tendency to increase with owners’ increasing personal experience with dogs, but decrease with increasing professional experience with dogs. As reading a dog’s body language and multimodal emotional behavior is likely to involve a social learning process, longer dog ownership may allow the owners to pick up on subtle changes in their dog’s behavior and to be familiar with (or sensitive to) different emotional triggers and contexts. This might enable them to report more (especially complex secondary) dog emotions. It could also be argued that this longer dog ownership would result in a stronger emotional bond between owner and dog with a concomitant increased risk of anthropomorphism and humanization bias toward their dogs [20,43], which may lead to an increased willingness to attribute human-like emotions to their dogs and an over-estimation of dogs’ emotional capabilities. On the other hand, longer professional experience with dogs may allow individual humans to be familiar with possible individual and/or breed variances in dog emotional behaviors triggered by the same emotional context, hence reducing the possibility of attributing different dog behaviors to different emotions and anthropomorphism bias toward their own dogs. However, most of our participants had a relatively long personal experience but relatively short professional experience with dogs. This skewed sample distribution might have biased our findings. These hypotheses could be systematically examined in future research. It should also be noted that these relationships between the number of reported dog emotions and owners’ personal/professional experiences were relatively weak, as they were not revealed by correlation analysis. Further research could re-examine these relationships by recruiting a larger cohort of dog owners. 

Furthermore, the number of reported cat emotions was reduced significantly if cat owners also had a dog at home (the results for dogs were similar regardless of any cats in the house). One interpretation is that cats may behave differently (e.g., being repressed and less expressive) if they live with dogs compared to if they live alone or with other cats. Owners may also report fewer cat emotions if they spend less time with the cat because the dog is always close by. However, it is worth mentioning that most dogs and cats can share a house peacefully, and tend to choose to spend time in the same room every day [44,45]. In addition to possible changes in cat behaviors, owners’ behaviors or attitudes may also lead to this variation in reported cat emotions. Perhaps owners of both species are overlooking cats’ emotional capabilities and/or are inferring dogs’ emotional states more frequently than cats because they believe that they have a greater level of emotional closeness with their dogs [35] and are more likely to treat dogs (but not cats) as ‘members of the family’ [20]. These hypotheses could also be systematically examined in future research.

It should be noted that the generalization of these reported research findings might be constrained by the limits of our sample population. For instance, the participating pet owners were dominated by female (87%) and Caucasian (90%) participants, as is common in such work. Given that the owners’ gender and cultural background may affect their ability to perceive dog emotions [19,24,25], future research could recruit a larger cohort of dog and cat owners, or one that is more focused on a more balanced gender and culture distribution, to systematically examine the impact of owner factors on the perception of animal emotions. 

Nevertheless, the variation in the number of emotions reported between species has several implications, both for animal welfare and human health and safety. One potential implication is that the emotional states of cats, particularly those living with dogs, are being overlooked or interpreted incorrectly. This could mean that the welfare needs of cats are not being met in terms of reducing the incidences of negative emotions (sadness, frustration, boredom, etc.) and increasing the opportunities to elicit positive emotions (happiness, amusement, etc.). If the findings are driven by owners having a relatively less positive belief in cats compared to dogs, this could also affect how they treat their cats (e.g., the misattribution of cat fear as anger may lead to the use of positive punishment, negatively impacting the cat’s welfare). Furthermore, being unable to interpret subtle cat behavioral cues may put an owner at risk. For example, petting-related aggression often occurs because owners miss those subtle cues indicating that the cat wants the owner to stop touching them [41,46].

Conversely, it is also possible that the emotional capabilities of dogs are being exaggerated and/or misinterpreted because of increased anthropomorphism and humanization trends in dog owners. Believing dogs are just another member of the family can lead to welfare issues. If owners do not appreciate how different dogs are, particularly concerning their communicative signals, they may interpret dog signals in a human-like way more frequently. For example, the reported sources of behavioral cues for detecting guilt/shame in dogs were ‘head posture’, ‘body posture’, and ‘eye contact’, with the most likely scenario being a dog that is holding its head low, contracting its body and avoiding eye contact. In fact, these behaviors are typically associated with a fearful, deferent, or submissive dog, and so scolding the dog when he exhibits these behaviors may exacerbate the dog’s fear/anxiety [9] and have a negative impact on its welfare. Humanization can also increase the risk to humans, as it increases the likelihood of people ignoring signs of discomfort or putting their dog in situations they cannot cope with, believing that their dog would never hurt them. For example, most dog bites involving children occur with familiar dogs when unsupervised in the home environment, and the behavior of the child toward the dog is the most common trigger [47].

## 5. Conclusions

This online study compared owners’ perceptions of their dogs’ and cats’ emotional expressions. Overall, more emotions were reported in dogs compared to cats, both from owners that owned just one species and those that owned both. While dogs and cats used a comparable set of behavioral signs to express the same emotion, distinct combinations tended to be associated with specific emotions in both cats and dogs. Although owner beliefs and anthropomorphisms are problematic in many situations, they are helpful as a starting point for an objective definition of animal emotions. With current technology, it is difficult to confirm animal emotional states; however, validating or refuting these owner beliefs could be the next step in this direction. This should be a high-priority research area as it is evident that regardless of whether owners are underestimating the emotional abilities of cats or overestimating that of dogs (or perhaps both), there are potentially important consequences for both humans and the animals they hope to care for.

## Figures and Tables

**Table 1:** Sources of behavioral cues were reported by over 50% of respondents for each emotion and species. Those that are common to both species are highlighted in bold.

Emotion	Dogs	Cats
Anger	**Facial expression**, **body posture**, tail height, **vocalisations**	**Facial expression,** ear posture, **body posture**, wagging tail, **vocalisations**
Disgust	**Facial expression**	**Facial expression**
Fear	Proximity to owner, facial expression, head posture, **ear posture**, **body posture, tail height**	**Ear posture, body posture, tail height**, speed of movement
Happiness/Joy	**Facial expression, head posture**, ear posture, **body posture, tail height**, wagging tail, **vocalisations,** speed of movement	**Facial expression, head posture, body posture, tail height, vocalisations**
Sadness	Facial expression, head posture, ear posture, **body posture**, tail height	**Body posture**
Surprise	**Facial expression**, head posture, ear posture, **body posture**	**Facial expression, body posture,** speed of movement
Anxiety	Proximity to owner, facial expression, ear posture, **body posture**, tail height	**Body posture**
Curiosity/Interest	**Facial expression, head posture, ear posture, body posture**, tail height	**Facial expression, head posture, ear posture, body posture**
Embarrassment	Facial expression, **body posture**	**Body posture**
Empathy	**Proximity to owner, physical contact**	**Proximity to owner, physical contact**
Envy/jealousy	Proximity to owner	None
Grief	Facial expression, body posture, physical contact	None
Guilt/shame	Head posture, body posture, eye contact	None
Love/affection	**Proximity to owner,** facial expression, **body posture**, eye contact, **physical contact**	**Proximity to owner**, head posture, **body posture**, vocalisations, **physical contact**
Pride	**Facial expression, head posture, body posture**	**Facial expression, head posture, body posture**
Amusement	Facial expression, wagging tail, vocalisations	Body posture
Boredom	None	None
Confusion	**Facial expression, head posture**	**Facial expression, head posture**
Disappointment	Facial expression, body posture	None
Frustration	**Vocalisations**	**Vocalisations**
Pain	Facial expression, **body posture**, vocalisations, **speed of movement**	**Body posture, speed of movement**
Positive anticipation	**Facial expression**, **head posture,** ear posture, **body posture**, tail height, wagging tail, **vocalisations**	**Facial expression, head posture, body posture, vocalisations**

**Table 2:** Pet ownership demographic details.

	Category	% of Dog Owners	% of Cat Owners
Personal experience with dogs or cats (Years)	2–10 years	43%	40%
	11–20 years	23%	22%
	21–30 years	15%	19%
	>30 years	18%	19%
Professional experience with dogs or cats	Yes	40%	24%
	No	60%	76%
Profession	Veterinary staff	23%	44%
	Trainer/Behaviourist	42%	8%
	Other (hands-on)	23%	31%
	Other	12%	18%
Professional experience (years)	<5 years	39%	46%
	5–10 years	34%	21%
	>10 years	27%	33%

**Table 3:** Dog demographic details.

Dog Demographic	Category	% of Dogs
Age	Junior (2–7 years old)	45%
	Senior (>7 years old)	55%
Sex	Female	48%
	Male	52%
Coat colour	Black	15%
	Brown/Tan	10%
	Cream/White	8%
	Golden/Red	12%
	Grey/Blue	2%
	Fur with 2 colours	37%
	Fur with 3+ colours	16%
Ear shape	Cropped	1%
	Drop/Folded	25%
	Pricked/Bat	17%
	Rose shaped	7%
	Semi-pricked/Button	20%
	V-shaped	30%
Muzzle length	Long	7%
	Medium	85%
	Short	8%
Tail shape	Docked/bobbed	9%
	Other	82%
	Tight curl	8%

**Table 4:** Cat demographic details.

Dog Demographic	Category	% of Cats
Age	Junior (2–7 years old)	37%
	Senior (>7 years old)	63%
Sex	Female	57%
	Male	43%
Coat length	Long hair	19%
	Short hair	81%
Coat pattern	Bi-color	30%
	Color point	2%
	Solid	20%
	Tabby	31%
	Tortoiseshell/Calico	17%
Ear shape	Pricked ears	98%
	Folded ears	2%
Tail shape	Bobbed	7%
	Curled tail	10%
	All others	83%

## Data Availability

Data are available from the authors.
